# 6-(4-Methyl­phen­yl)-2,10-dioxo-3,9-dioxa-6-aza-1(1,1′)-ferrocenacyclo­deca­phane

**DOI:** 10.1107/S1600536810043278

**Published:** 2010-11-06

**Authors:** Xin Leng, Bingqin Yang, Lin Cui, Bo Liu

**Affiliations:** a Key Laboratory of Synthetic and Natural Chemistry of the Ministry of Education, College of Chemistry and Materials Science, Northwest University of Xi’an, Taibai Bei Avenue 229, Xi’an 710069, Shaanxi, People’s Republic of China

## Abstract

In the title compound, [Fe(C_23_H_23_NO_4_)], the two cyclo­penta­dienyl (Cp) rings are nearly parallel, with a dihedral angle of 2.1 (1)°. The distance between the centroids of the Cp rings is 3.277 (8) Å. The relative orientation of the two Cp rings is characterized by a torsion angle of −64.3 (3)° defined by the two centroids and two substituted atoms.

## Related literature

For the definition of ferrocenophanes, see: Otón *et al.* (2005 ▶). For the properties of ferrocenophanes, see: Cayuela *et al.* (2004 ▶); Kulbaba & Manners (2001 ▶); Lu *et al.* (2006 ▶); Mizuta *et al.* (2003 ▶); Nguyen *et al.* (1999 ▶); Otón *et al.* (2006*a* ▶,*b* ▶); Suzaki *et al.* (2006 ▶). For a related structure, see: Gao *et al.* (2009 ▶). For the synthesis, see: Abd-Alla *et al.* (1993 ▶); Shivarkar *et al.* (2008 ▶). For a description of the Cambridge Structural Database, see: Allen (2002 ▶).
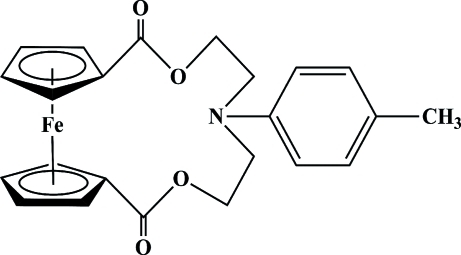


## Experimental

### 

#### Crystal data

[Fe(C_23_H_23_NO_4_)]
*M* *_r_* = 433.27
Orthorhombic, 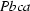

*a* = 15.028 (2) Å
*b* = 11.7488 (17) Å
*c* = 22.765 (3) Å
*V* = 4019.3 (10) Å^3^
*Z* = 8
Mo *K*α radiation
μ = 0.78 mm^−1^
*T* = 296 K
0.33 × 0.25 × 0.15 mm

#### Data collection

Bruker APEXII CCD diffractometer
Absorption correction: multi-scan (*SADABS*; Sheldrick, 1996 ▶) *T*_min_ = 0.784, *T*_max_ = 0.894
19079 measured reflections
3568 independent reflections
2357 reflections with *I* > 2σ(*I*)
*R*_int_ = 0.057

#### Refinement

*R*[*F* ^2^ > 2σ(*F* ^2^)] = 0.043
*wR*(*F* ^2^) = 0.128
*S* = 1.00
3568 reflections
263 parameters
H-atom parameters constrained
Δρ_max_ = 0.29 e Å^−3^
Δρ_min_ = −0.43 e Å^−3^

### 

Data collection: *APEX2* (Bruker, 2007 ▶); cell refinement: *SAINT* (Bruker, 2007 ▶); data reduction: *SAINT*; program(s) used to solve structure: *SHELXS97* (Sheldrick, 2008 ▶); program(s) used to refine structure: *SHELXL97* (Sheldrick, 2008 ▶); molecular graphics: *DIAMOND* (Brandenburg, 1999 ▶); software used to prepare material for publication: *SHELXL97*.

## Supplementary Material

Crystal structure: contains datablocks I, global. DOI: 10.1107/S1600536810043278/hy2361sup1.cif

Structure factors: contains datablocks I. DOI: 10.1107/S1600536810043278/hy2361Isup2.hkl

Additional supplementary materials:  crystallographic information; 3D view; checkCIF report

## Figures and Tables

**Fig. 1.**
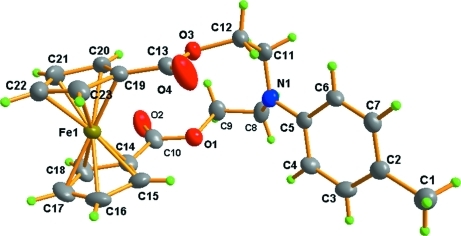
Molecular structure of the title compound. Displacement ellipsoids are drawn at the 50% probability level.

## supplementary crystallographic information

### Comment

Ferrocenophanes, in which the two cyclopentadienyl (Cp) rings are joined by an atomic or molecular bridge (Otón *et al.*, 2005), are found to be aromatic, highly stable and generally non-toxic, and have reversible redox characteristics (Mizuta *et al.*, 2003). In particular, ferrocenophanes are useful precursors to poly-ferrocenyl materials (Kulbaba & Manners, 2001; Nguyen *et al.*, 1999) and act as potential receptor towards cation or anion recognition (Cayuela *et al.*, 2004; Lu *et al.*, 2006; Suzaki *et al.*, 2006; Otón *et al.*, 2006*a*,*b*). As a part of our ongoing investigation of ferrocenophanes, the title compound has been prepared and we report here its crystal structure. Despite of the fact that structually characterized ferrocenophanes are well presented in the Cambridge Structural Database (Allen, 2002; Version 5.27, release February 2009), there is only one analogous compound structurally characterized (Gao *et al.*, 2009). From this viewpoint, X-ray single-crystal study of the title compound presents a certain descriptive interest.

The structure of the title compound is show in Fig. 1. The two Cp rings are nearly parallel, making a dihedral angle of 2.1 (1)°. The distance between the centroids of the Cp rings is 3.277 (8) Å. The angle formed between the two centroids and Fe1 is 177.67 (16)°. The relative orientation of the two Cp rings is characterized by the C10—*Cg*1—*Cg*2—C13 torsion angle of -64.3 (3)° (*Cg*1 and *Cg*2 are the centroids of C14–C18 ring and C19–C23 ring, respectively). The Fe—C distances range from 2.005 (3) to 2.045 (4) Å. The exocyclic bond lengths C13—C19 and C10—C14 are 1.453 (4) and 1.467 (5) Å, respectively.

### Experimental

2,2'-(*p*-Tolylazanediyl)diethanol was prepared as described by Shivarkar *et al.* (2008). 1,1'-Ferrocenedi(carbonyl chloride) was prepared as described by Abd-Alla *et al.* (1993). For the preparation of ferrocenophane, a mixture of 1,1'-ferrocenedi(carbonyl chloride) (2 g, 0.006 mol), dry dichloromethane (500 ml) and pyridine (1 ml, 0.012 mol) was stirred in dark. To this solution 2,2'-(*p*-tolylazanediyl)diethanol (0.006 mol) in dichloromethane was added dropwise over 1 h. The mixture was stirred for 24 h and then refluxed for another 8 h (monitored by TLC). The solvent was removed under atmospheric pressure. The residue was then purified by column chromatography on silica gel, eluting with ethyl acetate/petroleum ether (1:2) (yield: 38%). Melting point and NMR spectra confirmed identity and purity of the prepared compound.

Dark-red crystals of the title compound were obtained by slow concentration of a dichloromethane solution at room temperature.

### Refinement

All H atoms were positioned geometrically and refined using a riding model, with C—H = 0.93–0.98 Å and *U*_iso_(H) = 1.2(1.5 for methyl)*U*_eq_(C).

### Crystal data

**Table:**

[Fe(C_23_H_23_NO_4_)]	*D*_x_ = 1.432 Mg m^−^^3^
*M**_r_* = 433.27	Melting point: 407(8) K
Orthorhombic, *P**b**c**a*	Mo *K*α radiation, λ = 0.71073 Å
Hall symbol: -P 2ac 2ab	Cell parameters from 2477 reflections
*a* = 15.028 (2) Å	θ = 2.4–19.6°
*b* = 11.7488 (17) Å	µ = 0.78 mm^−^^1^
*c* = 22.765 (3) Å	*T* = 296 K
*V* = 4019.3 (10) Å^3^	Block, dark-red
*Z* = 8	0.33 × 0.25 × 0.15 mm
*F*(000) = 1808	

### Data collection

**Table:**

Bruker APEXII CCD diffractometer	3568 independent reflections
Radiation source: fine-focus sealed tube	2357 reflections with *I* > 2σ(*I*)
graphite	*R*_int_ = 0.057
φ and ω scans	θ_max_ = 25.1°, θ_min_ = 1.8°
Absorption correction: multi-scan (*SADABS*; Sheldrick, 1996)	*h* = −17→17
*T*_min_ = 0.784, *T*_max_ = 0.894	*k* = −14→12
19079 measured reflections	*l* = −27→24

### Refinement

**Table:**

Refinement on *F*^2^	Primary atom site location: structure-invariant direct methods
Least-squares matrix: full	Secondary atom site location: difference Fourier map
*R*[*F*^2^ > 2σ(*F*^2^)] = 0.043	Hydrogen site location: inferred from neighbouring sites
*wR*(*F*^2^) = 0.128	H-atom parameters constrained
*S* = 1.00	*w* = 1/[σ^2^(*F*_o_^2^) + (0.0623*P*)^2^ + 1.0856*P*] where *P* = (*F*_o_^2^ + 2*F*_c_^2^)/3
3568 reflections	(Δ/σ)_max_ < 0.001
263 parameters	Δρ_max_ = 0.29 e Å^−^^3^
0 restraints	Δρ_min_ = −0.43 e Å^−^^3^

### Fractional atomic coordinates and isotropic or equivalent isotropic displacement parameters (Å^2^)

**Table:**

	*x*	*y*	*z*	*U*_iso_*/*U*_eq_	
Fe1	0.12595 (3)	1.22967 (4)	0.052251 (19)	0.05457 (19)	
N1	0.00836 (17)	0.8972 (2)	0.20855 (11)	0.0599 (7)	
O1	0.16563 (14)	0.9967 (2)	0.15793 (9)	0.0653 (6)	
O2	0.26745 (17)	1.1325 (3)	0.17458 (12)	0.1046 (10)	
O3	−0.03453 (13)	1.13781 (17)	0.17171 (9)	0.0586 (6)	
O4	−0.11312 (18)	1.1282 (3)	0.08862 (13)	0.1183 (12)	
C1	−0.1716 (3)	0.5112 (3)	0.10871 (16)	0.0796 (11)	
H1A	−0.2031	0.4726	0.1395	0.119*	
H1B	−0.1285	0.4604	0.0918	0.119*	
H1C	−0.2128	0.5349	0.0790	0.119*	
C2	−0.1247 (2)	0.6140 (3)	0.13363 (14)	0.0589 (8)	
C3	−0.0345 (2)	0.6298 (3)	0.12735 (14)	0.0673 (9)	
H3	−0.0017	0.5761	0.1065	0.081*	
C4	0.0090 (2)	0.7224 (3)	0.15085 (14)	0.0629 (9)	
H4	0.0698	0.7308	0.1445	0.075*	
C5	−0.0357 (2)	0.8035 (3)	0.18383 (13)	0.0506 (7)	
C6	−0.1279 (2)	0.7883 (3)	0.18891 (14)	0.0598 (9)	
H6	−0.1613	0.8416	0.2096	0.072*	
C7	−0.1698 (2)	0.6964 (3)	0.16399 (14)	0.0616 (9)	
H7	−0.2312	0.6898	0.1679	0.074*	
C8	0.0986 (2)	0.8793 (3)	0.23124 (16)	0.0692 (10)	
H8A	0.1237	0.8120	0.2129	0.083*	
H8B	0.0955	0.8654	0.2732	0.083*	
C9	0.1598 (2)	0.9792 (3)	0.22014 (15)	0.0716 (10)	
H9A	0.1364	1.0469	0.2390	0.086*	
H9B	0.2183	0.9638	0.2362	0.086*	
C10	0.2191 (2)	1.0829 (4)	0.14117 (17)	0.0723 (10)	
C11	−0.0434 (2)	0.9829 (3)	0.23940 (14)	0.0659 (9)	
H11A	−0.0035	1.0287	0.2631	0.079*	
H11B	−0.0844	0.9449	0.2658	0.079*	
C12	−0.0951 (2)	1.0596 (3)	0.20000 (15)	0.0644 (9)	
H12A	−0.1262	1.0150	0.1706	0.077*	
H12B	−0.1388	1.1015	0.2227	0.077*	
C13	−0.0522 (2)	1.1668 (3)	0.11655 (15)	0.0624 (9)	
C14	0.2097 (2)	1.1067 (3)	0.07820 (16)	0.0707 (10)	
C15	0.1458 (3)	1.0613 (3)	0.03878 (16)	0.0754 (11)	
H15	0.1035	0.9998	0.0470	0.091*	
C16	0.1557 (4)	1.1210 (4)	−0.01512 (17)	0.0932 (14)	
H16	0.1197	1.1093	−0.0505	0.112*	
C17	0.2232 (3)	1.2012 (4)	−0.0088 (2)	0.0953 (14)	
H17	0.2425	1.2553	−0.0390	0.114*	
C18	0.2579 (3)	1.1935 (4)	0.04834 (19)	0.0934 (14)	
H18	0.3063	1.2396	0.0647	0.112*	
C19	0.0083 (2)	1.2533 (3)	0.09438 (14)	0.0550 (8)	
C20	0.0773 (3)	1.3066 (3)	0.12548 (15)	0.0713 (10)	
H20	0.0950	1.2909	0.1661	0.086*	
C21	0.1165 (3)	1.3873 (3)	0.0874 (2)	0.0986 (14)	
H21	0.1668	1.4368	0.0972	0.118*	
C22	0.0727 (3)	1.3847 (4)	0.0337 (2)	0.0963 (14)	
H22	0.0866	1.4318	−0.0006	0.116*	
C23	0.0055 (3)	1.3034 (4)	0.03710 (17)	0.0786 (11)	
H23	−0.0360	1.2832	0.0056	0.094*	

### Atomic displacement parameters (Å^2^)

**Table:**

	*U*^11^	*U*^22^	*U*^33^	*U*^12^	*U*^13^	*U*^23^
Fe1	0.0526 (3)	0.0578 (3)	0.0533 (3)	0.0040 (2)	0.0076 (2)	−0.0010 (2)
N1	0.0557 (16)	0.0556 (17)	0.0683 (17)	0.0009 (13)	−0.0075 (13)	0.0035 (14)
O1	0.0595 (13)	0.0737 (17)	0.0626 (15)	0.0009 (12)	−0.0063 (11)	0.0061 (12)
O2	0.0675 (17)	0.139 (3)	0.107 (2)	−0.0402 (17)	−0.0299 (16)	0.040 (2)
O3	0.0619 (13)	0.0543 (14)	0.0596 (13)	−0.0074 (10)	0.0036 (10)	0.0053 (11)
O4	0.084 (2)	0.181 (4)	0.090 (2)	−0.056 (2)	−0.0187 (16)	0.031 (2)
C1	0.086 (3)	0.076 (3)	0.077 (3)	−0.006 (2)	−0.001 (2)	−0.006 (2)
C2	0.060 (2)	0.060 (2)	0.0558 (19)	0.0012 (17)	−0.0036 (15)	−0.0005 (16)
C3	0.061 (2)	0.074 (3)	0.066 (2)	0.0120 (18)	0.0029 (16)	−0.0155 (18)
C4	0.0422 (17)	0.079 (2)	0.068 (2)	0.0032 (17)	0.0019 (15)	−0.0058 (19)
C5	0.0497 (17)	0.0499 (19)	0.0522 (17)	0.0038 (15)	0.0003 (14)	0.0093 (15)
C6	0.0491 (18)	0.057 (2)	0.073 (2)	0.0069 (16)	0.0139 (16)	0.0020 (17)
C7	0.0476 (18)	0.063 (2)	0.074 (2)	−0.0057 (16)	0.0036 (16)	0.0035 (18)
C8	0.066 (2)	0.066 (2)	0.076 (2)	−0.0091 (18)	−0.0226 (18)	0.0185 (19)
C9	0.068 (2)	0.080 (3)	0.067 (2)	−0.0086 (19)	−0.0201 (18)	0.0114 (19)
C10	0.0417 (18)	0.095 (3)	0.081 (3)	0.006 (2)	0.0005 (18)	0.018 (2)
C11	0.076 (2)	0.063 (2)	0.059 (2)	−0.0052 (18)	0.0068 (17)	0.0044 (17)
C12	0.0600 (19)	0.054 (2)	0.079 (2)	0.0017 (17)	0.0174 (17)	0.0076 (18)
C13	0.0496 (19)	0.074 (2)	0.064 (2)	0.0004 (17)	0.0087 (17)	−0.0032 (18)
C14	0.053 (2)	0.086 (3)	0.073 (2)	0.0204 (18)	0.0110 (18)	0.011 (2)
C15	0.097 (3)	0.062 (2)	0.067 (2)	0.024 (2)	0.006 (2)	−0.0123 (19)
C16	0.128 (4)	0.096 (3)	0.056 (2)	0.047 (3)	0.011 (2)	−0.013 (2)
C17	0.085 (3)	0.117 (4)	0.084 (3)	0.034 (3)	0.035 (2)	0.020 (3)
C18	0.053 (2)	0.128 (4)	0.100 (3)	0.010 (2)	0.017 (2)	0.041 (3)
C19	0.0543 (18)	0.054 (2)	0.0570 (19)	0.0107 (15)	0.0090 (15)	0.0019 (15)
C20	0.094 (3)	0.057 (2)	0.063 (2)	−0.011 (2)	0.020 (2)	−0.0145 (17)
C21	0.123 (4)	0.060 (3)	0.113 (4)	−0.029 (2)	0.040 (3)	−0.019 (3)
C22	0.128 (4)	0.061 (3)	0.100 (3)	0.018 (3)	0.046 (3)	0.014 (2)
C23	0.071 (2)	0.089 (3)	0.075 (2)	0.031 (2)	0.0124 (19)	0.022 (2)

### Geometric parameters (Å, °)

**Table:**

Fe1—C14	2.005 (3)	C7—H7	0.9300
Fe1—C21	2.022 (4)	C8—C9	1.512 (4)
Fe1—C15	2.024 (4)	C8—H8A	0.9700
Fe1—C18	2.030 (4)	C8—H8B	0.9700
Fe1—C19	2.031 (3)	C9—H9A	0.9700
Fe1—C20	2.033 (3)	C9—H9B	0.9700
Fe1—C22	2.034 (4)	C10—C14	1.467 (5)
Fe1—C23	2.036 (4)	C11—C12	1.489 (4)
Fe1—C17	2.045 (4)	C11—H11A	0.9700
Fe1—C16	2.045 (4)	C11—H11B	0.9700
N1—C5	1.402 (4)	C12—H12A	0.9700
N1—C11	1.453 (4)	C12—H12B	0.9700
N1—C8	1.467 (4)	C13—C19	1.453 (4)
O1—C10	1.348 (4)	C14—C15	1.418 (5)
O1—C9	1.434 (4)	C14—C18	1.424 (5)
O2—C10	1.203 (4)	C15—C16	1.421 (5)
O3—C13	1.328 (4)	C15—H15	0.9800
O3—C12	1.445 (4)	C16—C17	1.392 (6)
O4—C13	1.204 (4)	C16—H16	0.9800
C1—C2	1.509 (5)	C17—C18	1.405 (6)
C1—H1A	0.9600	C17—H17	0.9800
C1—H1B	0.9600	C18—H18	0.9800
C1—H1C	0.9600	C19—C20	1.404 (5)
C2—C7	1.368 (5)	C19—C23	1.432 (5)
C2—C3	1.377 (4)	C20—C21	1.413 (5)
C3—C4	1.377 (4)	C20—H20	0.9800
C3—H3	0.9300	C21—C22	1.389 (6)
C4—C5	1.387 (4)	C21—H21	0.9800
C4—H4	0.9300	C22—C23	1.392 (6)
C5—C6	1.401 (4)	C22—H22	0.9800
C6—C7	1.374 (4)	C23—H23	0.9800
C6—H6	0.9300		
			
C14—Fe1—C21	126.0 (2)	C8—C9—H9A	110.0
C14—Fe1—C15	41.22 (15)	O1—C9—H9B	110.0
C21—Fe1—C15	164.88 (19)	C8—C9—H9B	110.0
C14—Fe1—C18	41.32 (15)	H9A—C9—H9B	108.4
C21—Fe1—C18	106.1 (2)	O2—C10—O1	123.1 (3)
C15—Fe1—C18	69.19 (19)	O2—C10—C14	125.7 (4)
C14—Fe1—C19	120.38 (14)	O1—C10—C14	111.3 (3)
C21—Fe1—C19	68.10 (15)	N1—C11—C12	114.0 (3)
C15—Fe1—C19	109.48 (15)	N1—C11—H11A	108.7
C18—Fe1—C19	154.15 (15)	C12—C11—H11A	108.7
C14—Fe1—C20	107.74 (16)	N1—C11—H11B	108.7
C21—Fe1—C20	40.79 (15)	C12—C11—H11B	108.7
C15—Fe1—C20	127.75 (15)	H11A—C11—H11B	107.6
C18—Fe1—C20	118.71 (18)	O3—C12—C11	109.0 (3)
C19—Fe1—C20	40.42 (13)	O3—C12—H12A	109.9
C14—Fe1—C22	162.5 (2)	C11—C12—H12A	109.9
C21—Fe1—C22	40.05 (18)	O3—C12—H12B	109.9
C15—Fe1—C22	154.39 (19)	C11—C12—H12B	109.9
C18—Fe1—C22	124.2 (2)	H12A—C12—H12B	108.3
C19—Fe1—C22	68.48 (15)	O4—C13—O3	123.7 (3)
C20—Fe1—C22	68.32 (17)	O4—C13—C19	123.8 (3)
C14—Fe1—C23	156.13 (16)	O3—C13—C19	112.5 (3)
C21—Fe1—C23	67.3 (2)	C15—C14—C18	108.2 (4)
C15—Fe1—C23	121.41 (18)	C15—C14—C10	127.7 (4)
C18—Fe1—C23	161.81 (16)	C18—C14—C10	123.6 (4)
C19—Fe1—C23	41.21 (13)	C15—C14—Fe1	70.1 (2)
C20—Fe1—C23	68.28 (16)	C18—C14—Fe1	70.3 (2)
C22—Fe1—C23	40.00 (17)	C10—C14—Fe1	119.1 (2)
C14—Fe1—C17	68.53 (16)	C14—C15—C16	106.8 (4)
C21—Fe1—C17	118.0 (2)	C14—C15—Fe1	68.7 (2)
C15—Fe1—C17	68.40 (18)	C16—C15—Fe1	70.4 (2)
C18—Fe1—C17	40.33 (16)	C14—C15—H15	126.6
C19—Fe1—C17	165.00 (18)	C16—C15—H15	126.6
C20—Fe1—C17	152.5 (2)	Fe1—C15—H15	126.6
C22—Fe1—C17	106.63 (18)	C17—C16—C15	108.8 (4)
C23—Fe1—C17	126.15 (17)	C17—C16—Fe1	70.1 (2)
C14—Fe1—C16	68.52 (16)	C15—C16—Fe1	68.8 (2)
C21—Fe1—C16	152.1 (2)	C17—C16—H16	125.6
C15—Fe1—C16	40.88 (16)	C15—C16—H16	125.6
C18—Fe1—C16	67.8 (2)	Fe1—C16—H16	125.6
C19—Fe1—C16	129.04 (19)	C16—C17—C18	108.8 (4)
C20—Fe1—C16	166.29 (19)	C16—C17—Fe1	70.1 (2)
C22—Fe1—C16	119.29 (18)	C18—C17—Fe1	69.3 (2)
C23—Fe1—C16	109.46 (18)	C16—C17—H17	125.6
C17—Fe1—C16	39.81 (17)	C18—C17—H17	125.6
C5—N1—C11	119.0 (3)	Fe1—C17—H17	125.6
C5—N1—C8	117.8 (3)	C17—C18—C14	107.4 (4)
C11—N1—C8	115.1 (3)	C17—C18—Fe1	70.4 (2)
C10—O1—C9	115.1 (3)	C14—C18—Fe1	68.4 (2)
C13—O3—C12	117.3 (3)	C17—C18—H18	126.3
C2—C1—H1A	109.5	C14—C18—H18	126.3
C2—C1—H1B	109.5	Fe1—C18—H18	126.3
H1A—C1—H1B	109.5	C20—C19—C23	107.3 (3)
C2—C1—H1C	109.5	C20—C19—C13	126.8 (3)
H1A—C1—H1C	109.5	C23—C19—C13	125.9 (3)
H1B—C1—H1C	109.5	C20—C19—Fe1	69.85 (18)
C7—C2—C3	116.4 (3)	C23—C19—Fe1	69.60 (18)
C7—C2—C1	121.7 (3)	C13—C19—Fe1	127.8 (2)
C3—C2—C1	121.9 (3)	C19—C20—C21	107.3 (4)
C2—C3—C4	122.2 (3)	C19—C20—Fe1	69.73 (18)
C2—C3—H3	118.9	C21—C20—Fe1	69.2 (2)
C4—C3—H3	118.9	C19—C20—H20	126.3
C3—C4—C5	121.6 (3)	C21—C20—H20	126.3
C3—C4—H4	119.2	Fe1—C20—H20	126.3
C5—C4—H4	119.2	C22—C21—C20	109.1 (4)
C4—C5—C6	115.8 (3)	C22—C21—Fe1	70.4 (2)
C4—C5—N1	121.8 (3)	C20—C21—Fe1	70.0 (2)
C6—C5—N1	122.3 (3)	C22—C21—H21	125.4
C7—C6—C5	121.3 (3)	C20—C21—H21	125.4
C7—C6—H6	119.3	Fe1—C21—H21	125.4
C5—C6—H6	119.3	C21—C22—C23	108.0 (4)
C2—C7—C6	122.5 (3)	C21—C22—Fe1	69.5 (2)
C2—C7—H7	118.7	C23—C22—Fe1	70.1 (2)
C6—C7—H7	118.7	C21—C22—H22	126.0
N1—C8—C9	113.1 (3)	C23—C22—H22	126.0
N1—C8—H8A	109.0	Fe1—C22—H22	126.0
C9—C8—H8A	109.0	C22—C23—C19	108.2 (4)
N1—C8—H8B	109.0	C22—C23—Fe1	69.9 (2)
C9—C8—H8B	109.0	C19—C23—Fe1	69.19 (18)
H8A—C8—H8B	107.8	C22—C23—H23	125.9
O1—C9—C8	108.3 (3)	C19—C23—H23	125.9
O1—C9—H9A	110.0	Fe1—C23—H23	125.9
			
C7—C2—C3—C4	0.8 (5)	C19—Fe1—C18—C17	172.7 (3)
C1—C2—C3—C4	−178.8 (3)	C20—Fe1—C18—C17	−156.7 (3)
C2—C3—C4—C5	2.0 (5)	C22—Fe1—C18—C17	−74.5 (4)
C3—C4—C5—C6	−3.3 (5)	C23—Fe1—C18—C17	−48.3 (8)
C3—C4—C5—N1	179.1 (3)	C16—Fe1—C18—C17	36.7 (3)
C11—N1—C5—C4	176.1 (3)	C21—Fe1—C18—C14	126.7 (3)
C8—N1—C5—C4	−36.8 (4)	C15—Fe1—C18—C14	−38.1 (2)
C11—N1—C5—C6	−1.4 (4)	C19—Fe1—C18—C14	53.8 (5)
C8—N1—C5—C6	145.7 (3)	C20—Fe1—C18—C14	84.4 (3)
C4—C5—C6—C7	1.9 (5)	C22—Fe1—C18—C14	166.6 (3)
N1—C5—C6—C7	179.5 (3)	C23—Fe1—C18—C14	−167.2 (5)
C3—C2—C7—C6	−2.2 (5)	C17—Fe1—C18—C14	−118.9 (4)
C1—C2—C7—C6	177.4 (3)	C16—Fe1—C18—C14	−82.2 (3)
C5—C6—C7—C2	0.8 (5)	O4—C13—C19—C20	176.1 (4)
C5—N1—C8—C9	142.6 (3)	O3—C13—C19—C20	−1.8 (5)
C11—N1—C8—C9	−69.0 (4)	O4—C13—C19—C23	−0.9 (6)
C10—O1—C9—C8	−179.3 (3)	O3—C13—C19—C23	−178.8 (3)
N1—C8—C9—O1	−60.2 (4)	O4—C13—C19—Fe1	−91.8 (4)
C9—O1—C10—O2	9.3 (5)	O3—C13—C19—Fe1	90.2 (3)
C9—O1—C10—C14	−170.6 (3)	C14—Fe1—C19—C20	81.7 (3)
C5—N1—C11—C12	−74.8 (4)	C21—Fe1—C19—C20	−38.2 (2)
C8—N1—C11—C12	137.3 (3)	C15—Fe1—C19—C20	125.9 (2)
C13—O3—C12—C11	144.0 (3)	C18—Fe1—C19—C20	43.6 (5)
N1—C11—C12—O3	−73.6 (4)	C22—Fe1—C19—C20	−81.4 (3)
C12—O3—C13—O4	−3.1 (5)	C23—Fe1—C19—C20	−118.3 (3)
C12—O3—C13—C19	174.9 (2)	C17—Fe1—C19—C20	−155.0 (6)
O2—C10—C14—C15	−171.0 (4)	C16—Fe1—C19—C20	167.7 (2)
O1—C10—C14—C15	8.8 (5)	C14—Fe1—C19—C23	−160.0 (3)
O2—C10—C14—C18	0.0 (6)	C21—Fe1—C19—C23	80.2 (3)
O1—C10—C14—C18	179.9 (3)	C15—Fe1—C19—C23	−115.8 (3)
O2—C10—C14—Fe1	−84.6 (4)	C18—Fe1—C19—C23	161.9 (4)
O1—C10—C14—Fe1	95.3 (4)	C20—Fe1—C19—C23	118.3 (3)
C21—Fe1—C14—C15	169.0 (2)	C22—Fe1—C19—C23	36.9 (3)
C18—Fe1—C14—C15	−118.8 (3)	C17—Fe1—C19—C23	−36.7 (7)
C19—Fe1—C14—C15	85.3 (3)	C16—Fe1—C19—C23	−74.0 (3)
C20—Fe1—C14—C15	127.6 (2)	C14—Fe1—C19—C13	−39.8 (4)
C22—Fe1—C14—C15	−158.3 (5)	C21—Fe1—C19—C13	−159.7 (4)
C23—Fe1—C14—C15	51.3 (5)	C15—Fe1—C19—C13	4.4 (3)
C17—Fe1—C14—C15	−81.3 (3)	C18—Fe1—C19—C13	−77.9 (5)
C16—Fe1—C14—C15	−38.4 (3)	C20—Fe1—C19—C13	−121.5 (4)
C21—Fe1—C14—C18	−72.2 (3)	C22—Fe1—C19—C13	157.1 (4)
C15—Fe1—C14—C18	118.8 (3)	C23—Fe1—C19—C13	120.2 (4)
C19—Fe1—C14—C18	−155.9 (3)	C17—Fe1—C19—C13	83.5 (7)
C20—Fe1—C14—C18	−113.6 (3)	C16—Fe1—C19—C13	46.1 (4)
C22—Fe1—C14—C18	−39.5 (6)	C23—C19—C20—C21	−0.6 (4)
C23—Fe1—C14—C18	170.1 (4)	C13—C19—C20—C21	−178.0 (3)
C17—Fe1—C14—C18	37.5 (3)	Fe1—C19—C20—C21	59.2 (3)
C16—Fe1—C14—C18	80.4 (3)	C23—C19—C20—Fe1	−59.8 (2)
C21—Fe1—C14—C10	46.1 (4)	C13—C19—C20—Fe1	122.7 (3)
C15—Fe1—C14—C10	−122.9 (4)	C14—Fe1—C20—C19	−116.3 (2)
C18—Fe1—C14—C10	118.3 (5)	C21—Fe1—C20—C19	118.7 (4)
C19—Fe1—C14—C10	−37.6 (4)	C15—Fe1—C20—C19	−75.0 (3)
C20—Fe1—C14—C10	4.7 (4)	C18—Fe1—C20—C19	−160.0 (2)
C22—Fe1—C14—C10	78.8 (6)	C22—Fe1—C20—C19	81.8 (3)
C23—Fe1—C14—C10	−71.5 (6)	C23—Fe1—C20—C19	38.6 (2)
C17—Fe1—C14—C10	155.8 (4)	C17—Fe1—C20—C19	166.3 (3)
C16—Fe1—C14—C10	−161.3 (4)	C16—Fe1—C20—C19	−44.5 (8)
C18—C14—C15—C16	0.1 (4)	C14—Fe1—C20—C21	125.0 (3)
C10—C14—C15—C16	172.2 (3)	C15—Fe1—C20—C21	166.3 (3)
Fe1—C14—C15—C16	60.3 (2)	C18—Fe1—C20—C21	81.4 (3)
C18—C14—C15—Fe1	−60.3 (3)	C19—Fe1—C20—C21	−118.7 (4)
C10—C14—C15—Fe1	111.9 (4)	C22—Fe1—C20—C21	−36.8 (3)
C21—Fe1—C15—C14	−36.2 (7)	C23—Fe1—C20—C21	−80.0 (3)
C18—Fe1—C15—C14	38.2 (2)	C17—Fe1—C20—C21	47.6 (5)
C19—Fe1—C15—C14	−114.2 (2)	C16—Fe1—C20—C21	−163.1 (7)
C20—Fe1—C15—C14	−72.6 (3)	C19—C20—C21—C22	0.2 (5)
C22—Fe1—C15—C14	165.1 (3)	Fe1—C20—C21—C22	59.7 (3)
C23—Fe1—C15—C14	−158.3 (2)	C19—C20—C21—Fe1	−59.6 (2)
C17—Fe1—C15—C14	81.6 (3)	C14—Fe1—C21—C22	165.4 (2)
C16—Fe1—C15—C14	118.0 (4)	C15—Fe1—C21—C22	−165.8 (6)
C14—Fe1—C15—C16	−118.0 (4)	C18—Fe1—C21—C22	124.5 (3)
C21—Fe1—C15—C16	−154.3 (6)	C19—Fe1—C21—C22	−82.2 (3)
C18—Fe1—C15—C16	−79.8 (3)	C20—Fe1—C21—C22	−120.0 (4)
C19—Fe1—C15—C16	127.8 (3)	C23—Fe1—C21—C22	−37.5 (2)
C20—Fe1—C15—C16	169.4 (3)	C17—Fe1—C21—C22	82.7 (3)
C22—Fe1—C15—C16	47.1 (5)	C16—Fe1—C21—C22	51.6 (5)
C23—Fe1—C15—C16	83.7 (3)	C14—Fe1—C21—C20	−74.6 (3)
C17—Fe1—C15—C16	−36.4 (3)	C15—Fe1—C21—C20	−45.8 (8)
C14—C15—C16—C17	−0.4 (4)	C18—Fe1—C21—C20	−115.5 (3)
Fe1—C15—C16—C17	58.8 (3)	C19—Fe1—C21—C20	37.8 (2)
C14—C15—C16—Fe1	−59.2 (2)	C22—Fe1—C21—C20	120.0 (4)
C14—Fe1—C16—C17	−81.8 (3)	C23—Fe1—C21—C20	82.5 (3)
C21—Fe1—C16—C17	45.5 (5)	C17—Fe1—C21—C20	−157.3 (3)
C15—Fe1—C16—C17	−120.5 (4)	C16—Fe1—C21—C20	171.6 (4)
C18—Fe1—C16—C17	−37.2 (3)	C20—C21—C22—C23	0.3 (5)
C19—Fe1—C16—C17	165.8 (2)	Fe1—C21—C22—C23	59.8 (3)
C20—Fe1—C16—C17	−158.4 (7)	C20—C21—C22—Fe1	−59.5 (3)
C22—Fe1—C16—C17	80.8 (3)	C14—Fe1—C22—C21	−42.7 (6)
C23—Fe1—C16—C17	123.6 (3)	C15—Fe1—C22—C21	171.5 (3)
C14—Fe1—C16—C15	38.7 (2)	C18—Fe1—C22—C21	−73.3 (3)
C21—Fe1—C16—C15	166.0 (4)	C19—Fe1—C22—C21	81.1 (3)
C18—Fe1—C16—C15	83.4 (3)	C20—Fe1—C22—C21	37.5 (2)
C19—Fe1—C16—C15	−73.7 (3)	C23—Fe1—C22—C21	119.1 (4)
C20—Fe1—C16—C15	−37.9 (9)	C17—Fe1—C22—C21	−113.9 (3)
C22—Fe1—C16—C15	−158.7 (3)	C16—Fe1—C22—C21	−155.1 (3)
C23—Fe1—C16—C15	−115.9 (3)	C14—Fe1—C22—C23	−161.8 (4)
C17—Fe1—C16—C15	120.5 (4)	C21—Fe1—C22—C23	−119.1 (4)
C15—C16—C17—C18	0.6 (5)	C15—Fe1—C22—C23	52.4 (5)
Fe1—C16—C17—C18	58.6 (3)	C18—Fe1—C22—C23	167.6 (3)
C15—C16—C17—Fe1	−58.0 (3)	C19—Fe1—C22—C23	−38.0 (2)
C14—Fe1—C17—C16	81.8 (3)	C20—Fe1—C22—C23	−81.6 (3)
C21—Fe1—C17—C16	−157.8 (3)	C17—Fe1—C22—C23	127.0 (3)
C15—Fe1—C17—C16	37.3 (3)	C16—Fe1—C22—C23	85.7 (3)
C18—Fe1—C17—C16	120.2 (4)	C21—C22—C23—C19	−0.6 (4)
C19—Fe1—C17—C16	−47.4 (8)	Fe1—C22—C23—C19	58.8 (2)
C20—Fe1—C17—C16	169.1 (3)	C21—C22—C23—Fe1	−59.4 (3)
C22—Fe1—C17—C16	−116.0 (3)	C20—C19—C23—C22	0.7 (4)
C23—Fe1—C17—C16	−76.6 (3)	C13—C19—C23—C22	178.3 (3)
C14—Fe1—C17—C18	−38.4 (3)	Fe1—C19—C23—C22	−59.2 (3)
C21—Fe1—C17—C18	82.0 (4)	C20—C19—C23—Fe1	60.0 (2)
C15—Fe1—C17—C18	−82.9 (3)	C13—C19—C23—Fe1	−122.5 (3)
C19—Fe1—C17—C18	−167.6 (6)	C14—Fe1—C23—C22	166.6 (4)
C20—Fe1—C17—C18	48.9 (5)	C21—Fe1—C23—C22	37.5 (2)
C22—Fe1—C17—C18	123.8 (3)	C15—Fe1—C23—C22	−156.3 (3)
C23—Fe1—C17—C18	163.2 (3)	C18—Fe1—C23—C22	−34.6 (7)
C16—Fe1—C17—C18	−120.2 (4)	C19—Fe1—C23—C22	119.6 (4)
C16—C17—C18—C14	−0.6 (5)	C20—Fe1—C23—C22	81.7 (3)
Fe1—C17—C18—C14	58.6 (3)	C17—Fe1—C23—C22	−71.4 (3)
C16—C17—C18—Fe1	−59.2 (3)	C16—Fe1—C23—C22	−112.7 (3)
C15—C14—C18—C17	0.3 (4)	C14—Fe1—C23—C19	46.9 (5)
C10—C14—C18—C17	−172.2 (3)	C21—Fe1—C23—C19	−82.1 (2)
Fe1—C14—C18—C17	−59.8 (3)	C15—Fe1—C23—C19	84.0 (3)
C15—C14—C18—Fe1	60.2 (2)	C18—Fe1—C23—C19	−154.3 (6)
C10—C14—C18—Fe1	−112.4 (3)	C20—Fe1—C23—C19	−37.9 (2)
C14—Fe1—C18—C17	118.9 (4)	C22—Fe1—C23—C19	−119.6 (4)
C21—Fe1—C18—C17	−114.4 (3)	C17—Fe1—C23—C19	169.0 (3)
C15—Fe1—C18—C17	80.8 (3)	C16—Fe1—C23—C19	127.6 (3)
